# Association of the HALP Score with Dyslipidemia: A Large, Nationwide Retrospective Study

**DOI:** 10.3390/medicina59112002

**Published:** 2023-11-14

**Authors:** Yazeed Alshuweishi, Ahmed M. Basudan, Mohammed Alfaifi, Hussam Daghistani, Mohammad A. Alfhili

**Affiliations:** 1 Chair of Medical and Molecular Genetics Research, Department of Clinical Laboratory Sciences, College of Applied Medical Sciences, King Saud University, Riyadh 12372, Saudi Arabia; yalshuweishi@ksu.edu.sa (Y.A.);; 2 Department of Clinical Laboratory Sciences, College of Applied Medical Sciences, King Khalid University, Abha 61421, Saudi Arabia; 3 Department of Clinical Biochemistry, Faculty of Medicine, King Abdulaziz University, Jeddah 21589, Saudi Arabia; hmdaghistani@kau.edu.sa; 4 Department of Clinical Pathology, Al Borg Diagnostics, Jeddah 23437, Saudi Arabia

**Keywords:** HALP score, dyslipidemia, cardiovascular disease, biomarker, Saudi Arabia

## Abstract

*Background and Objectives*: Dyslipidemia is a major risk factor for cardiovascular disease (CVD). The identification of new biomarkers that may enhance the risk assessment of lipid abnormalities is a promising approach in improving risk prediction of CVD. There is no information on the association of the hemoglobin, albumin, lymphocyte, and platelet (HALP) score with dyslipidemia. The aim of this study was to investigate the clinical utility of the HALP score in light of dyslipidemia. *Materials and Methods:* A retrospective analysis of 7192 subjects was initiated to assess the association between the HALP score and disturbed lipid markers. Medians were compared by Mann–Whitney U or Kruskal–Wallis tests and the diagnostic performance and risk assessment were calculated. *Results:* Median HALP score among all subjects was 53.3, with varying values between males and females. Notably, median HALP was significantly elevated in all forms of dyslipidemia and among males and females irrespective of age. The odds of having elevated HALP score values were significantly higher in all lipid abnormalities. Moreover, HALP score was significantly yet weakly correlated with lipid markers, while the highest diagnostic accuracy of the HALP score was observed with an elevated ratio of total cholesterol to high-density lipoprotein (TC/HDL) (area under the curve, AUC = 0.6411, *p* < 0.0001). The decision curve analysis (DCA) showed that the HALP score can reliably predict the presence of dyslipidemia. *Conclusions:* This study demonstrates that the HALP score is a novel, cost-effective index that is associated with a disturbed lipid profile. Further investigation of the nature of this association is needed.

## 1. Introduction

The imbalance of lipids, including triglycerides, high-density lipoprotein (HDL), low-density lipoprotein (LDL), and cholesterol, is known as dyslipidemia (DLD). This disorder can be caused by poor diet, hypertension, smoking, or hereditary predisposition and can lead to atherosclerosis, a dominant cause of cardiovascular disease and early death [1]. Atherosclerotic disease is the leading cause of death worldwide and in Saudi Arabia [2,3]. It was shown that the prevalence of hypercholesterolemia in Saudi people was 56.7% and 43.3% for men and women, respectively [4]. Additionally, the prevalence of dyslipidemia among Saudi adults varies from 20% to 44%, with aberrant triglycerides at the top [5]. The primary tool for assessing dyslipidemia is the fasting lipid panel that includes total cholesterol, LDL, HDL, and triglycerides. There is dispute regarding the appropriate age to start screening for dyslipidemia [6]. Some guidelines suggest universal screening should be undertaken in all children, with the rationale that early identification allows for earlier interventions [7]. Alternatively, a targeted approach to screen only subjects with a family history of lipid disorders has been suggested as a cost-effective approach, yet this approach failed to identify up to 60% of at-risk children [8,9].

The onset and progression of dyslipidemia is multifactorial and often associated with increased caloric intake, physical inactivity, and excessive stimulation of proinflammatory cytokine production [10,11]. There are number of disagreements in various studies with respect to variability of hematological parameters in patients with dyslipidemia, with the majority of studies showing higher hemoglobin levels which may indicate a higher risk of atherosclerosis, previously associated with an unfavorable lipid profile [12,13,14,15,16]. Serum albumin is considered an important marker in reflecting nutritional status as well as an indicator of the severity of illness in several diseases [17,18]. Moreover, excessive pro-inflammatory cytokines can result in detrimental effects on several metabolic tissues including adipose tissue, skeletal muscle, and liver. Particularly, inflammatory pathway activation in visceral adipose tissue observed in obesity is associated with decreased lipogenic markers, likely as a result of more cells switching to an inflammatory phenotype than a lipid storage phenotype [19]. This primarily causes ectopic lipid accumulation in skeletal muscle and liver which eventually dampens peripheral insulin signaling leading to obesity-related metabolic disorders such as systemic insulin resistance, hyperlipidemia, and type 2 diabetes [20]. Moreover, platelets can participate in immune response and inflammation by secreting immune modulators which are chemotactic for neutrophils, monocytes, and lymphocytes [21].

Lipid disturbance, chronic inflammation, and malnutrition are common in patients with cardiovascular diseases [22,23]. The hemoglobin, albumin, lymphocyte, and platelet (HALP) score has emerged as an immunonutritional biomarker that involves a group of routinely examined indicators to provide a single composite score that represents the overall health status of patients [24]. These four parameters are essential considerations for immune and nutritional status where combining these parameters predicts the prognosis of the inflammatory status more accurately than using a single index alone. It was initially developed by Chen et al. to predict gastric carcinoma prognosis, and it is calculated by {hemoglobin (g/L) × albumin (g/L) × lymphocytes (/L)}/platelets (/L) [25]. This score has been found to be a good prognostic indicator for various types of cancer, including colorectal cancer, gastric, and hepatocellular carcinoma [26,27,28]. In these conditions, the HALP score was significantly reduced along with poor immune response. This suggests that HALP score represents a valuable tool for clinicians to assess a patient’s inflammatory status.

Although HALP has emerged in the literature as a new prognostic biomarker for poor immune response in immunodeficiency conditions, the levels of HALP score and its clinical utility have not been examined in conditions associated with chronic inflammation that create overactive inflammatory responses such as dyslipidemia, hypertension, and obesity. Furthermore, there are no studies that have examined the baseline HALP levels within a large, representative sample of the Saudi population. Therefore, the aim of this study is to characterize the patterns of HALP score across a large Saudi population sample and the influence of abnormal lipid profile on this score. Moreover, this study will determine the prognostic value of the HALP score and its association estimates with abnormal lipid markers and ratios in different age and sex groups.

## 2. Materials and Methods

### 2.1. Study Design and Data Collection

**Figure 1:**
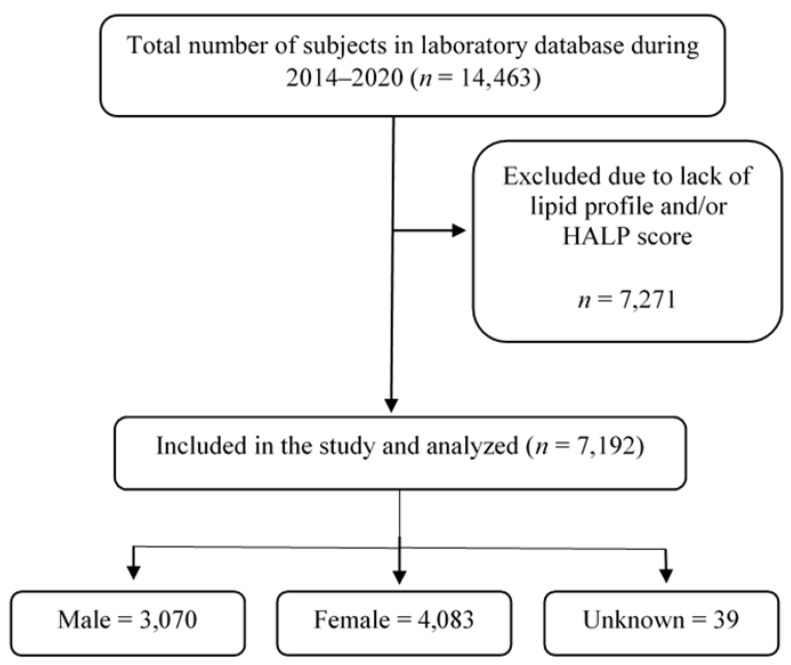
A flow chart of study design.

The presented data in this study were obtained from Al Borg Diagnostics and the study protocol was approved by the Biomedical Ethics Unit at Al Borg Diagnostics (approval number: 07/21, approved on 27 December 2021). Age, gender, and laboratory data between 2015 to 2020 were retrieved from the Al Borg Diagnostics database. As shown in Figure 1, out of a total of 14,463 potential subjects, only 7192 subjects were included based on the availability of laboratory records of both a complete HALP score component (hemoglobin, lymphocyte, albumin, and platelet) as well as complete lipid profiles (HDL, LDL, total cholesterol, and triglycerides). Males and females were separated and age groups were formed as shown in Table 1.

The study subjects were categorized as young (<18 years), young adults (18–39 years), adults (40–64 years), and elderlies (≥65 years). Dyslipidemia (DLD) was defined by a TC of ≥200 mg/dL, LDL of ≥130 mg/dL, HDL of <40 mg/dL, or TG of ≥150 mg/dL, TC/HDL ratio of ≥6, an LDL/HDL ratio of >2.5, and a TG/HDL ratio of >2 [29]. Accordingly, normolipidemia (NLD) was defined by a TC of <200 mg/dL, LDL of <130 mg/dL, HDL of ≥40 mg/dL, TG of <150 mg/dL.TC/HDL ratio of <6, an LDL/HDL ratio of ≤2.5, and a TG/HDL ratio of ≤2. HALP score was calculated according to the following formula: {hemoglobin (g/L) × albumin (g/L) × lymphocytes (/L)}/platelets (/L) [30]. According to the receiver operating characteristics (ROC) curve analysis, the optimal cut-off value for HALP score was >58.59 based on the highest AUC values obtained with TC/HDL, and thus a cut-off value greater than 58.59 was considered a high HALP score.

### 2.2. Statistics

As revealed by the D’Agostino and Pearson test and Kolmogorov–Smirnov test (*p* < 0.0001), the data were not normally distributed and therefore non-parametric tests were used. Results were compared either by the unpaired, Mann–Whitney U test for two groups or one-way ANOVA with the Kruskal–Wallis test followed by Dunn’s multiple comparisons test for three or more groups and then displayed as medians ± interquartile range (IQR). Associations between lipid parameters and HALP score were assessed by simple linear regression analysis, and risk assessment was determined by calculations of the prevalence risk (PR) and odds ratio (OR). The potential value of HALP score for discriminating abnormal lipid profile was measured by ROC curve analysis and area under the curve (AUC) determination. A DCA curve and calibration plot were created by the “dcurves” and “predtools” packages, respectively, using R statistical environment [31]. GraphPad Prism v9.2.0 (GraphPad Software, Inc., San Diego, CA, USA) was used for statistical analysis, and statistical significance was set at *p* < 0.05.

## 3. Results

### 3.1. Baseline Levels of HALP Score among Study Subjects

As shown in Table 1, the total population included in this study was 7192 subjects with a median HALP score of 53.30 (±39.04–70.75). The study subjects were stratified based on the values of HALP score into normal HALP group (≤58.59) and high HALP group (>58.59). The characteristics of the study subjects are presented in Table 2. All baseline parameters had significant differences (all *p* values < 0.0001) among groups of HALP score, except for testosterone and thyroid-stimulating hormone (TSH).

**Figure 2:**
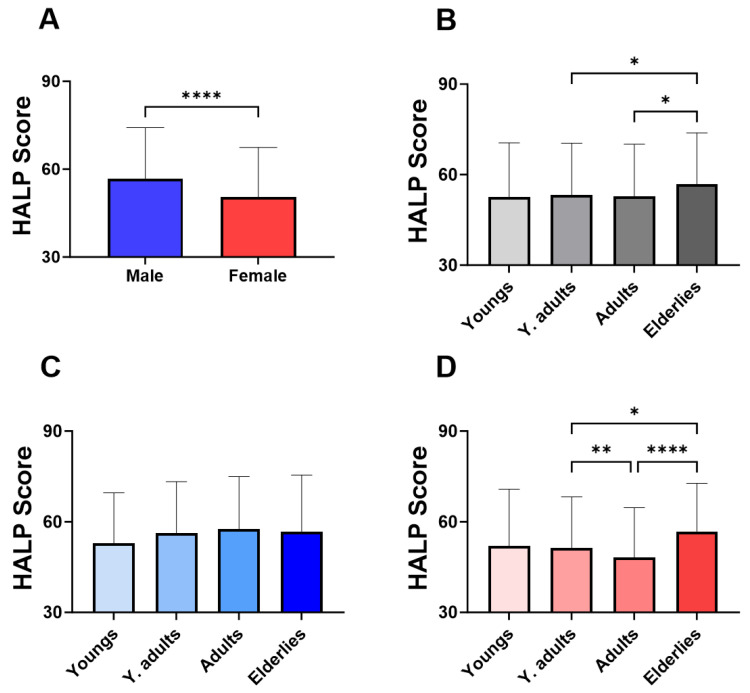
**Effect of gender and age on the baseline HALP score.** Medians ± IQR of HALP score values in (**A**) male and female, (**B**) in age groups of both genders, (**C**) of males, and (**D**) of females. * (*p* < 0.05), ** (*p* < 0.01), and **** (*p* < 0.0001) indicate significant difference.

Gender-specific analysis revealed that the median value of males was 56.68 (±59.86–62.02) which was approximately 10% higher than the median of HALP observed in females (Figure 2A; 50.58 ± 54.01–55.61). When HALP score was determined across several age groups as shown in Figure 2B, elderlies had the highest value of HALP score (56.81 ± 57.55–61.45) compared to adults (52.81 ± 56.23–58.36) and young adults (53.20 ± 56.04–57.95). However, when only males were considered, no significant differences were observed among age groups (Figure 2C). However, the HALP score level among female age groups was similar to the one observed when both genders were considered (Figure 2D).

### 3.2. Higher Level of HALP Score Is Observed with Elevated Lipid Markers Irrespective of Age and Gender

**Figure 3:**
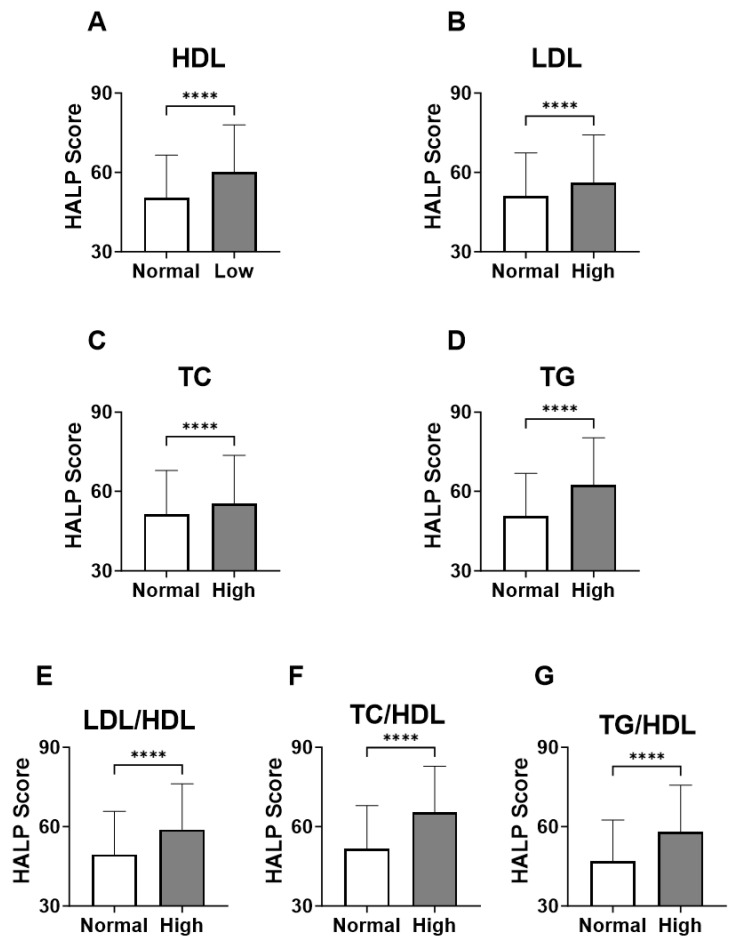
**HALP score among individual lipid markers in both genders.** Medians ± IQR of (**A**) HDL, (**B**) LDL, (**C**) TC, (**D**) TG, (**E**) LDL/HDL, (**F**) TC/HDL, and (**G**) TG/HDL. **** (*p* < 0.0001) indicate significant difference.

**Figure 4:**
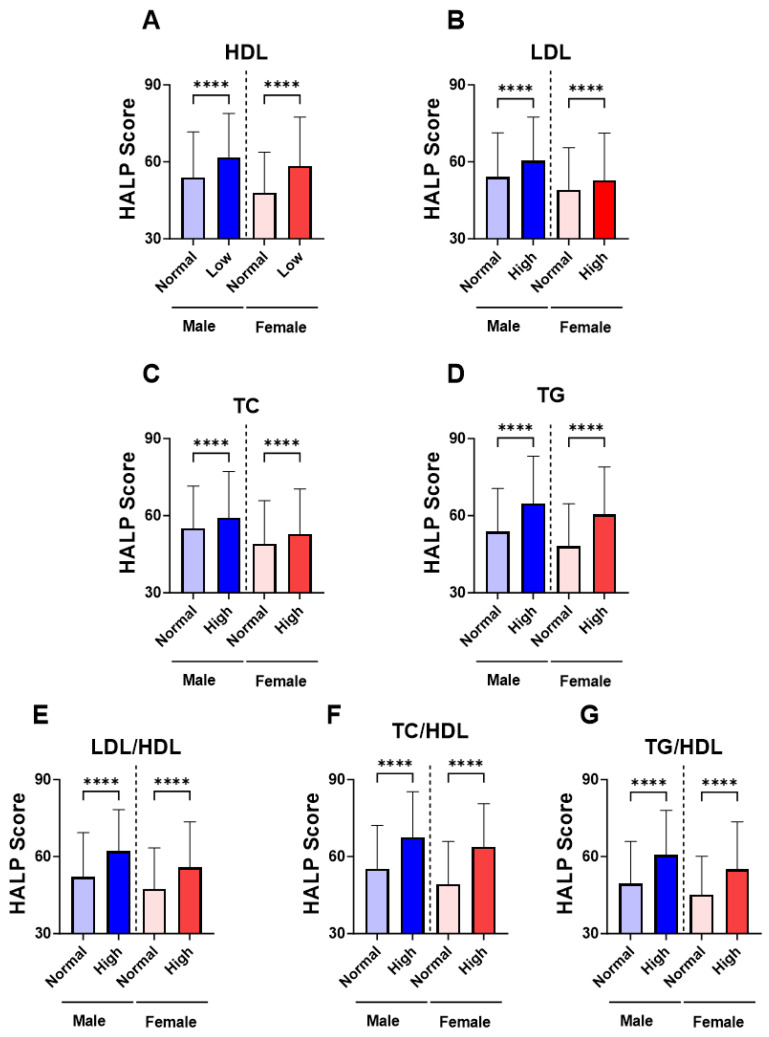
**Gender-specific levels of HALP score among individual lipid markers.** Medians ± IQR of (**A**) HDL, (**B**) LDL, (**C**) TC, (**D**) TG, (**E**) LDL/HDL, (**F**) TC/HDL, and (**G**) TG/HDL. **** (*p* < 0.0001) indicate significant difference.

**Figure 5:**
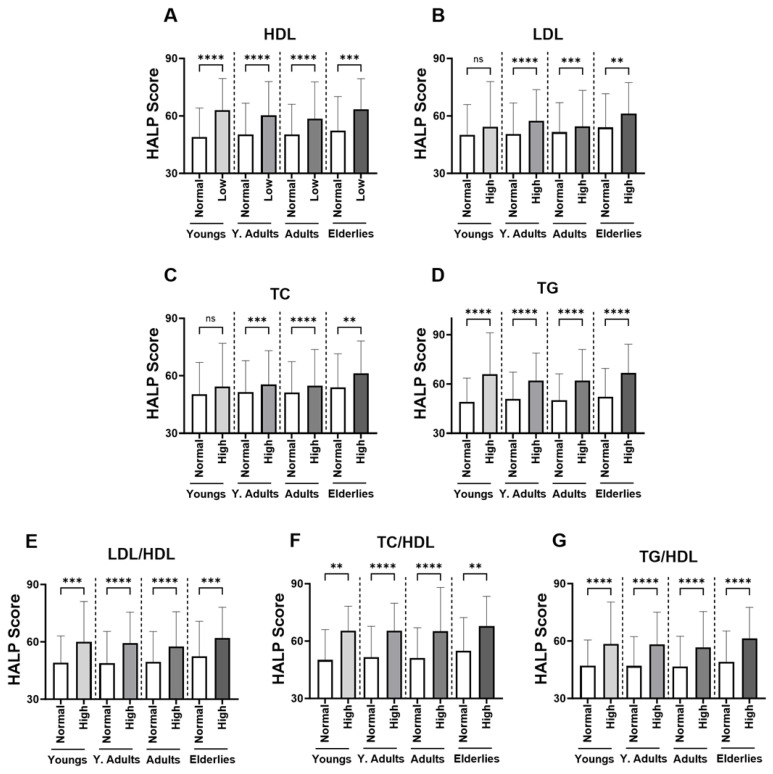
**Effect of age on the HALP score among individual lipid markers in both genders.** Medians ± IQR of (**A**) HDL, (**B**) LDL, (**C**) TC, (**D**) TG, (**E**) LDL/HDL, (**F**) TC/HDL, and (**G**) TG/HDL. ** (*p* < 0.01), *** (*p* < 0.001), and **** (*p* < 0.0001) indicate significant difference, while ns indicates no significance.

In Figure 3, groups were formed based on the normal and abnormal levels of the individual marker of lipid profile in order to examine the relationship between HALP score values and adverse lipid profile. Our analysis revealed a significant increase in the level of HALP score in the low-HDL subjects (Figure 3A; 60.12 ± 62.09–64.63) compared to the normal HDL group (Figure 2A; 50.43 ± 53.91–55.39). Likewise, in Figure 3B,C, subjects with high LDL or TC had a significantly increased HALP score in comparison to those with normal LDL or TC (LDL; 56.20 ± 59.21–61.20 vs. 51.02 ± 54.39–56.10) (TC; 55.49 ± 58.60–60.54 vs. 51.37 ± 54.95–56.70). The increase in HALP score was clearly apparent in the high-TG group (62.49 ± 64.75–67.80) compared to the normal TG group (50.58 ± 53.97–55.36) (Figure 3D). Similarly, HALP score was also elevated in high lipid ratios including LDL/HDL (Figure 3E; 58.92 ± 61.41–63.55 vs. 49.43± 52.77–54.34), TC/HDL (Figure 3F; 65.53 ± 66.50–70.90 vs. 51.62 ± 55.08–56.41) and TG/HDL (Figure 3G; 58.02 ± 60.88–62.66 vs. 47.04± 49.92–51.69). These patterns of lipid profiles were also observed when males and females when considered separately (Figure 4A–G). Moreover, age-specific analysis revealed that HALP score in young subjects was only unchanged in high LDL and high TC (Figure 5B,C).

### 3.3. Correlation of HALP Score with Lipid Profile Markers

**Figure 6:**
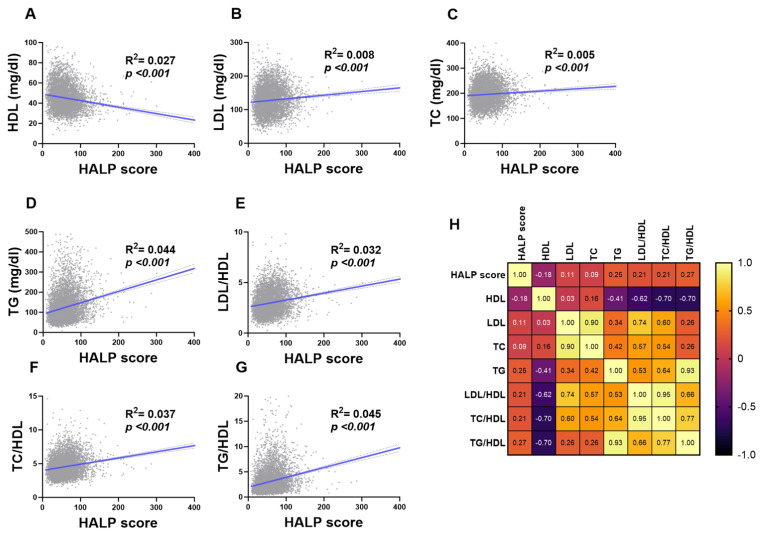
**Association of HALP score with lipid markers in both genders.** Simple linear regression of the association between HALP score and (**A**) HDL, (**B**) LDL, (**C**) TC, (**D**) TG, (**E**) LDL/HDL, (**F**) TC/HDL, and (**G**) TG/HDL. (**H**) A correlation matrix with correlation coefficients is also shown.

Figure 6 demonstrated the association between the individual components of lipid markers and HALP score in the studied population: (A) HDL, (B) LDL, (C) TC, (D) TG, (E) LDL/HDL, (F) TC/HDL, and (G) TG/HDL. The HALP score showed a positive trend (or negative trend with HDL) within each component of lipid profiles (*p* < 0.001).

**Figure 7:**
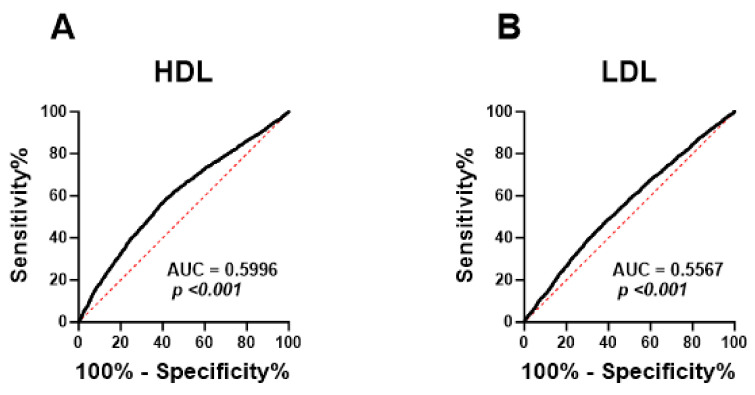

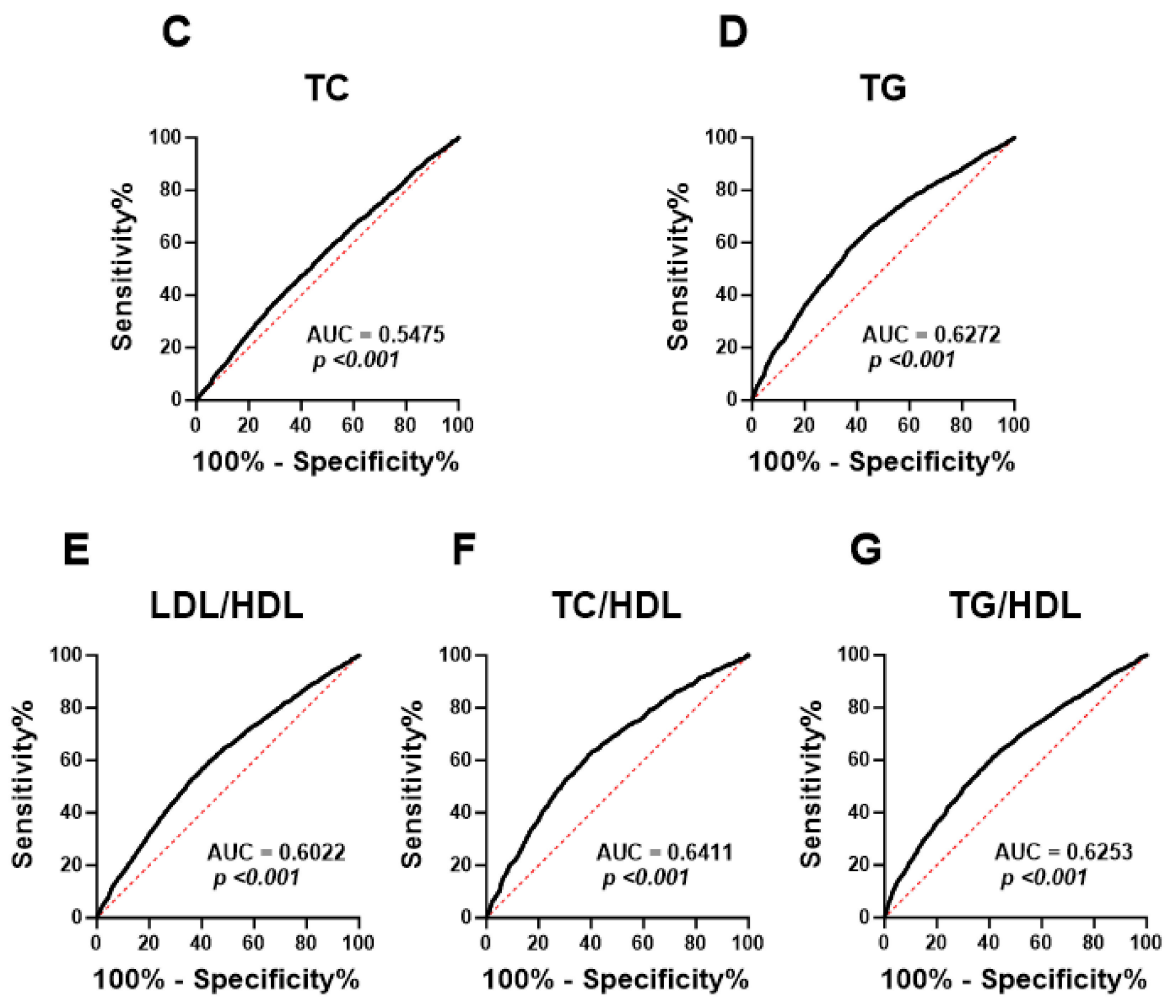
**ROC curve analysis of the diagnostic accuracy of HALP score for lipid markers in both genders.** AUC and *p* values of the diagnostic accuracy of HDL (**A**), LDL (**B**), TC (**C**), TG (**D**), LDL/HDL (**E**), TC/HDL (**F**), and TG/HDL (**G**).

Next, ROC curve analysis was performed in order to evaluate the ability of HALP score to distinguish subjects with normal lipid markers and those with lipid abnormalities. HALP exhibited poor diagnostic ability for low HDL (Figure 7A; AUC = 0.5996, *p* < 0.001), high LDL (Figure 7B; AUC = 0.5567, *p* < 0.001), high TC (Figure 7C; AUC = 0.5475, *p* < 0.001) and LDL/HDL (Figure 7E; AUC = 0.6022, *p* < 0.001). However, HALP showed a better performance in discriminating high TG (Figure 7D; AUC = 0.6272, *p* < 0.001), TC/HDL (Figure 7F; AUC = 0.6411, *p* < 0.001) and TG/HDL (Figure 7G; AUC = 0.6253, *p* < 0.001).

### 3.4. Prevalence and Risk Assessment of HALP Score in Light of Dyslipidemia

To measure the incidence of various forms of dyslipidemia relative to HALP score, a cut-off HALP value (>58.59) with the highest sensitivity and specificity to detect disturbed lipid profiles was selected. As shown in Table 3, the prevalence of abnormal HDL was higher when the HALP score was considered high. Furthermore, elevated levels of LDL, TC, TG, LDL/HDL, TC/HDL, and TG/HDL was more prevalent in subjects with a high HALP score.

To assess the risk of HALP associated with abnormality in lipid markers, we measured PR and OR values as shown in Table 4. Elevated HALP score values were found to be a risk factor for all abnormal lipid markers. In particular, elevated TG/HDL had the greatest PR value (1.64, *p* < 0.0001). Consistent with prevalence findings, OR values showed that the odds of having a HALP score above the designated cut-off were significantly higher with an elevated lipid marker (Table 4).

### 3.5. Predictive Power of HALP Score for Dyslipidemia

**Figure 8:**
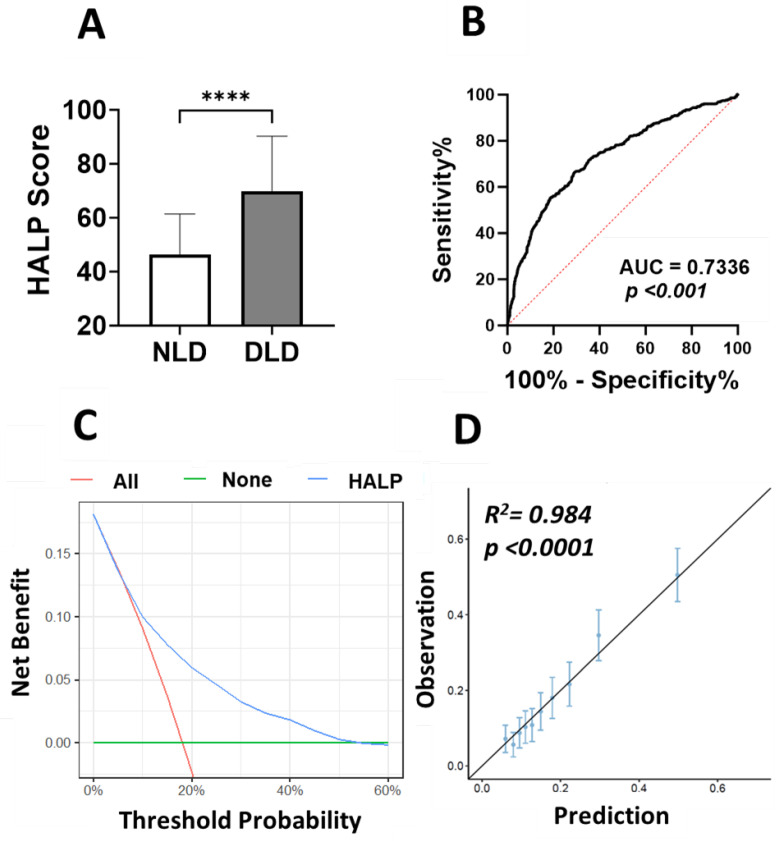
**DCA of HALP score prediction model to estimate DLD.** (**A**) HALP score in NLD and dyslipidemic DLD subjects, **** (*p* < 0.0001) indicate significant difference. (**B**) ROC curve analysis of the diagnostic accuracy of HALP score for discriminating DLD from NLD. (**C**) Decision-making curve for the HALP score model is shown in blue where the *x*-axis represents the risk threshold probabilities and the *y*-axis indicates the net benefit. (**D**) Calibration plot of HALP score. The diagonal line represents a perfect prediction by an ideal model. Each dot represents a decile of the observed vs. predicted value. The vertical bars around each dot represent 95% CI.

Here we examined the prognostic value of HALP score in light of lipid profile. Subjects were grouped as normolipidemic (NLD) and dyslipidemic (DLD) as defined in the study design section (Section 2.1). The HALP score was significantly higher in the DLD group (Figure 8A) and the AUC value based on ROC curve analysis was 0.734. Next, we estimated the relationship between HALP score and dyslipidemia via logistic regression and found that HALP score was highly significantly associated with the dyslipidemia as outcome [OR 27.9 ± 17.1–46.2; *p* < 0.001)]. Decision curve analysis examined the potential benefit of using the HALP score as a clinical utility for dyslipidemia (DLD) risk prediction. The net clinical benefit of using the HALP score model was compared to “treat-all” and “treat-none” criteria to compute the net benefit of the model. DLD incidence and HALP score values for the study subjects were converted to predicted probabilities using logistic regression and used to construct the decision curve. The net clinical benefit in light of DLD was beneficial for a reasonable range of threshold probabilities between 7% and 52% and showed the greatest net benefit in this range (Figure 8C). In addition, a calibration plot was generated as a visual tool to assess the agreement between predictions and observations in different percentiles (deciles) of the predicted values. Dyslipidemia (DLD) and HALP score were used to build a prediction model where the subjects were divided into 10 groups by using the deciles of the predicted probability of the fitted logistic model. In this model, there was high agreement between the predicted and observed probabilities across different percentiles, as shown in Figure 8D (R^2^ = 0.984, *p* < 0.0001).

## 4. Discussion

Dyslipidemia is largely a silent disease and often remains undiagnosed with no presenting symptoms [32]. Thus, predictive biomarkers from easily accessible specimens, such as blood, are of great importance to healthcare providers in order to improve the early diagnosis or prognostic monitoring of dyslipidemia. This study investigated the HALP score in a population-based sample, observing its association and diagnostic accuracy in light of dyslipidemia. The main finding is that the HALP score was significantly elevated in all forms of lipid abnormalities. This pattern persisted across all age and sex groups as demonstrated in our age- and gender-specific analyses. To the best of our knowledge, our study is the first of its kind to investigate the association between the HALP score and dyslipidemia in Saudi Arabia.

Initially, this study examined the existence of gender disparity at the baseline level of HALP score. Our findings showed that males were 56.68, which was a 10% higher level of HALP value compared to females at 50.58. Gender disparity observed with the baseline of HALP score is also observed in the US population [33]. These findings were not surprising since it is well known that males and females display differences in a number of reference laboratory values. For instance, hemoglobin concentration in women is approximately 12% lower than men [34]. Additionally, platelet count of healthy females is higher to that of healthy males [35]. Clinical data also indicate that inflammatory responses differ across sexes, where females have been shown to have a more robust inflammatory response compared to males [36]. These sex differences are largely linked to the multiple effects of sex hormones on several laboratory parameters [37,38,39]. Furthermore, age-dependant analysis showed that elderlies had higher levels of HALP score compared to young adult and adult groups. Given that lymphocyte and platelet, which are important components of HALP score, are known as inflammatory markers, this might explain the increase in HALP score in elderlies as inflammation increases with aging [40]. This study is the first to establish HALP ranges in different age and sex groups, potentially providing a basis for a uniform baseline of HALP values across Saudi population.

The findings in this study suggest that all forms of dyslipidemia are associated with increased HALP score, pointing at individual or combined alterations in any of the four indexes of HALP score, namely hemoglobin, albumin, lymphocyte, and platelet. Notably, this association between elevated HALP score and dyslipidemia was observed in males and females and almost across all age groups. While a difference in HALP score baseline may exist between males and females, the current results suggest that it does not significantly impact the potential utility of HALP score as biomarker for dyslipidemia. The underlying mechanism behind such association is likely related to the role of individual components of HALP score in light of lipid metabolism. Hemoglobin, one of HALP score indexes, has been associated with adverse lipid profile and it has been reported that hemoglobin concentration can be elevated by smoking, which is a recognized risk factor for dyslipidemia [41]. Higher hemoglobin levels were also seen in obesity, a well-known risk factor for cardiometabolic diseases [42]. With metabolic syndrome, higher hemoglobin levels have also been associated with NAFLD, dyslipidemia, and hypertension [43]. The mechanism behind the aforementioned reports might be related to increased blood viscosity with changes in blood volume, impaired endothelial cell function, or altered iron and ferritin levels [44,45,46]. The role of hyperhemoglobinemia as a risk factor for dyslipidemia and its comorbidities are not fully understood and warrant further research.

Hyperalbuminemia can cause an increase in HALP score values. The major determinant of albumin production under physiological conditions is energy supply in which malnutrition is associated with reduced serum albumin levels, whereas high serum albumin levels have been observed in overnutrition conditions such as obesity and metabolic syndrome [47]. Recently, Bae et al., reported that serum albumin was associated with higher levels of HOMA-IR, and the presence of IFG and NAFLD in 9029 subjects without diabetes [47]. These reports indicate that hyperlipidemia is associated with high albumin levels likely due to the influence of dyslipidemia-related insulin resistance on serum albumin levels. Furthermore, cellular blood components, lymphocyte, and platelet, are part of the HALP score index where any alterations in their levels are reflected on the HALP score values. Dyslipidemia has potential effects on lymphocyte subsets and platelet function. In a study involving 51 dyslipidemic patients and healthy controls, it was found that several lymphocyte subsets were significantly higher in dyslipidemic patients and significantly associated with hypercholesterolemia in peripheral blood [48]. On the other hand, hyperlipidemia contributes to platelet hyperactivity where oxidized LDL particles activate platelets, leading to morphological changes [49]. Based on the above reports regarding the individual effects of HALP components on lipid profile, we could infer that hemoglobin, albumin, and lymphocyte might be the unfavorable prognostic factors, but platelet might be the favorable one in the context of dyslipidemia.

In this study, we initially evaluated the correlation between HALP score and the abnormal changes in individual lipid parameters. The best diagnostic performance was observed with increased TG and TC/HDL. Particularly, a recent report proposed that TC/HDL provided additional information not available in more commonly used single lipid parameters [50]. In this report, subjects with higher TC/HDL, but normal LDL and non-HDL levels, had more atherosclerosis, elevated triglycerides, higher BMI, and greater prevalence of hypertension, diabetes, and smoking compared to those with normal TC/HDL. Furthermore, the results of the decision curve analysis (DCA) for the prediction model show that HALP score was beneficial and showed the greatest net benefit for a reasonable range of threshold probabilities between 7% and 52%. These findings suggest that HALP is not only a simple, rapid, and cost-effective marker but also could serve as a promising biomarker for monitoring and predicting the incidence and progression of lipid disorders.

The HALP score has the capacity of providing a comprehensive assessment of the nutritional state as well as inflammatory response of the body, with a simple, non-invasive, and cost-effective way. We have shown here in a large sample of the Saudi population that HALP score has a statistically significant association with disturbed lipid parameters in all forms of lipid abnormalities among males and females and across all age groups. A number of recent studies have proposed that HALP score is a useful prognostic biomarker in various diseases, including cancers, cerebrovascular, and cardiovascular diseases [51,52]. Notably, Leetanaporn et al. reported that HALP score ≤22.2 tended to have a lower age, lower comorbidity rate, and lower body mass index [53]. This suggests a role of inflammation and adiposity in the relationship of HALP score with lipid disturbance. Although the relationship between inflammation and dyslipidemia is not fully understood, epidemiological data showed that dyslipidemia patients exhibit a proinflammatory state that is characterized by higher cytokine levels, including TNF-α and IL-6 [54]. Particularly, the persistent arterial inflammation driven by hyperlipidemia, which results in the synthesis of cytokines that have the potential to trigger acute phase proteins like CRP and endothelial circulatory cellular adhesion molecules (CAMs), leading to endothelial dysfunction and the subsequent expression of inflammatory biomarkers [54].

The strength of the current study is based on the very high sample size, little analytical variability as a result of automated data collection and processing, examination of a full lipid panel, and the ability to determine prevalence and risk estimates. However, there were some limitations in this study. First, the causality of the association between the HALP score and dyslipidemia cannot be established as a cross-sectional study; we should undergo further prospective studies to confirm our findings. Additionally, there is a lack of information on confounding factors such as the body mass index, smoking, existing comorbidities, and past medication intake that may or may not have a major impact on the findings.

## 5. Conclusions

This work demonstrates that all forms of dyslipidemia are associated with a higher HALP score in the Saudi population. Thus, our findings call for further longitudinal studies to examine the relationship between the HALP score and dyslipidemia, and to evaluate the prognostic value of this score in the screening as well as management of dyslipidemia. Although the HALP score has been mostly studied in the context of cancer, future studies must investigate the patterns of change in the HALP score in other diseases and reach a better understanding of the clinical value of this inexpensive and readily available marker for the screening, diagnosis, and prognosis of various diseases.

## Figures and Tables

**Table 1:** Distribution of study subjects.

Gender	Number of Subjects (%)	Median HALP Score (±IQR)
ALL	7192	53.30 (±39.04–70.75)
Male	3070 (42.7)	56.68 (±42.24–74.20)
Young	181 (2.5)	52.98 (±41.71–69.63)
Young adults	1160 (16.1)	56.21 (±42.15–73.31)
Adults	1408 (19.6)	57.68 (±42.81–75.02)
Elderlies	321 (4.5)	56.81 (±40.59–75.47)
Female	4083 (56.8)	50.58 (±36.90–67.44)
Young	186 (2.6)	52.02 (±38.79–70.77)
Young adults	2055 (28.6)	51.43 (±36.88–68.27)
Adults	1490 (20.7)	52.02 (±35.92–64.69)
Elderlies	352 (4.9)	56.81 (±41.17–72.76)
Unknown	39 (0.5)	64.45 (±51.07–76.15)

**Table 2:** Characteristics of study subjects.

Parameters	Normal HALP	High HALP	*p*
WBC count (×10^3^/μL)	5.3 (±4.28–6.53)	6.365 (±5.22–7.55)	<0.0001
RBC count (×10^6^/μL)	5.03 (±4.71–5.45)	5.53 (±5.17–5.90)	<0.0001
FBG (mg/dL)	95 (±88–105)	98 (±90–113)	<0.0001
Hemoglobin (g/dL)	13.5 (±12.30–14.60)	15.5 (±14.40–16.50)	<0.0001
Albumin (g/dL)	4.1 (±3.9–4.4)	4.3 (±4.1–4.6)	<0.0001
Lymphocyte count (×10^3^/μL)	2.09 (±1.72–2.48)	2.72 (±2.32–3.20)	<0.0001
Platelet count (×10^3^/μL)	289 (±246–339)	234 (±202–269)	<0.0001
ALT (U/L)	18 (±13–26)	25 (±18–37)	<0.0001
AST (U/L)	18 (±15–23)	21 (±17–26)	<0.0001
Calcium (mg/dL)	9.5 (±9.2–9.9)	9.7 (±9.4–10.0)	<0.0001
Potassium (mEg/L)	4.4 (±4.1–4.6)	4.4 (±4.2–4.7)	<0.0001
Sodium (mg/dL)	139 (±138–141)	140 (±138–142)	<0.0001
Testosterone (ng/dL)	4.7 (±3.6–6.2)	4.6 (±3.6–6.0)	0.4337
TSH (mIU/L)	1.69 (±1.12–2.59)	1.69 (±1.14–2.45)	0.3582

Results are shown as medians (±IQR). WBC, white blood cell; RBC, red blood cell; FBG, fasting blood glucose; ALT, alanine aminotransferase; AST, aspartate aminotransferase; TSH, thyroid-stimulating hormone.

**Table 3:** Prevalence of HALP in dyslipidemia among the studied population.

	N-HDL	L-HDL
N-HALP	63.60	47.33
H-HALP	36.40	52.67
	N-LDL	H-LDL
N-HALP	62.38	53.37
H-HALP	37.62	46.63
	N-TC	H-TC
N-HALP	61.43	54.39
H-HALP	38.57	45.61
	N-TG	H-TG
N-HALP	63.28	42.82
H-HALP	36.72	57.18
	N-LDL/HDL	H-LDL/HDL
N-HALP	65.40	49.29
H-HALP	34.60	50.71
	N-TC/HDL	H-TC/HDL
N-HALP	61.36	38.64
H-HALP	38.64	61.36
	N-TG/HDL	H-TG/HDL
N-HALP	69.94	50.85
H-HALP	30.06	49.15

**Table 4:** Risk assessment of dyslipidemia based on the HALP score.

Parameter	PR	95% CI	Z Statistic	*p*	OR	95% CI	Z Statistic	*p*
HDL	1.45	1.37–0.15	13.53	*p* < 0.0001	1.94	1.76–2.15	13.01	*p* < 0.0001
LDL	1.24	1.17–1.31	7.73	*p* < 0.0001	1.45	1.32–1.59	7.70	*p* < 0.0001
TC	1.41	1.34–1.48	13.38	*p* < 0.0001	1.34	1.22–1.47	6.01	*p* < 0.0001
TG	1.56	1.48–1.64	16.19	*p* < 0.0001	2.30	2.06–2.57	14.86	*p* < 0.0001
LDL/HDL	1.47	1.39–1.55	13.73	*p* < 0.0001	1.95	1.77–2.14	13.69	*p* < 0.0001
TC/LDL	1.59	1.50–1.69	15.30	*p* < 0.0001	2.52	2.19–2.90	12.96	*p* < 0.0001
TG/LDL	1.64	1.53–1.74	15.10	*p* < 0.0001	2.25	2.03–2.49	15.89	*p* < 0.0001

## Data Availability

Data are available from the corresponding author upon reasonable request, and with permission of Al Borg Diagnostics.
